# Supplementary figures and images for: Intraspecific and spatial variation in habitat use by sperm whales (Physeter macrocephalus) along the west coast of Martinique
Source: PeerJ. 2025 Jul 14;13:e19614. doi: 10.7717/peerj.19614 (PMC12269786; doi:10.7717/peerj.19614)

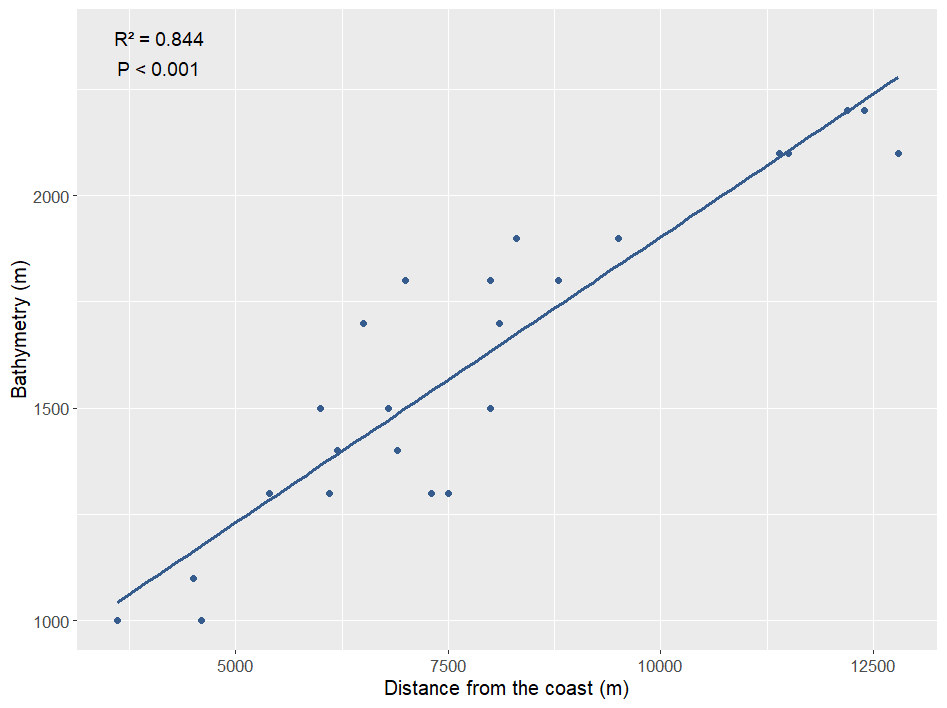

Supplement: Supplemental Information 12 [file peerj-13-19614-s012.zip › figure/Figure_S2.png]

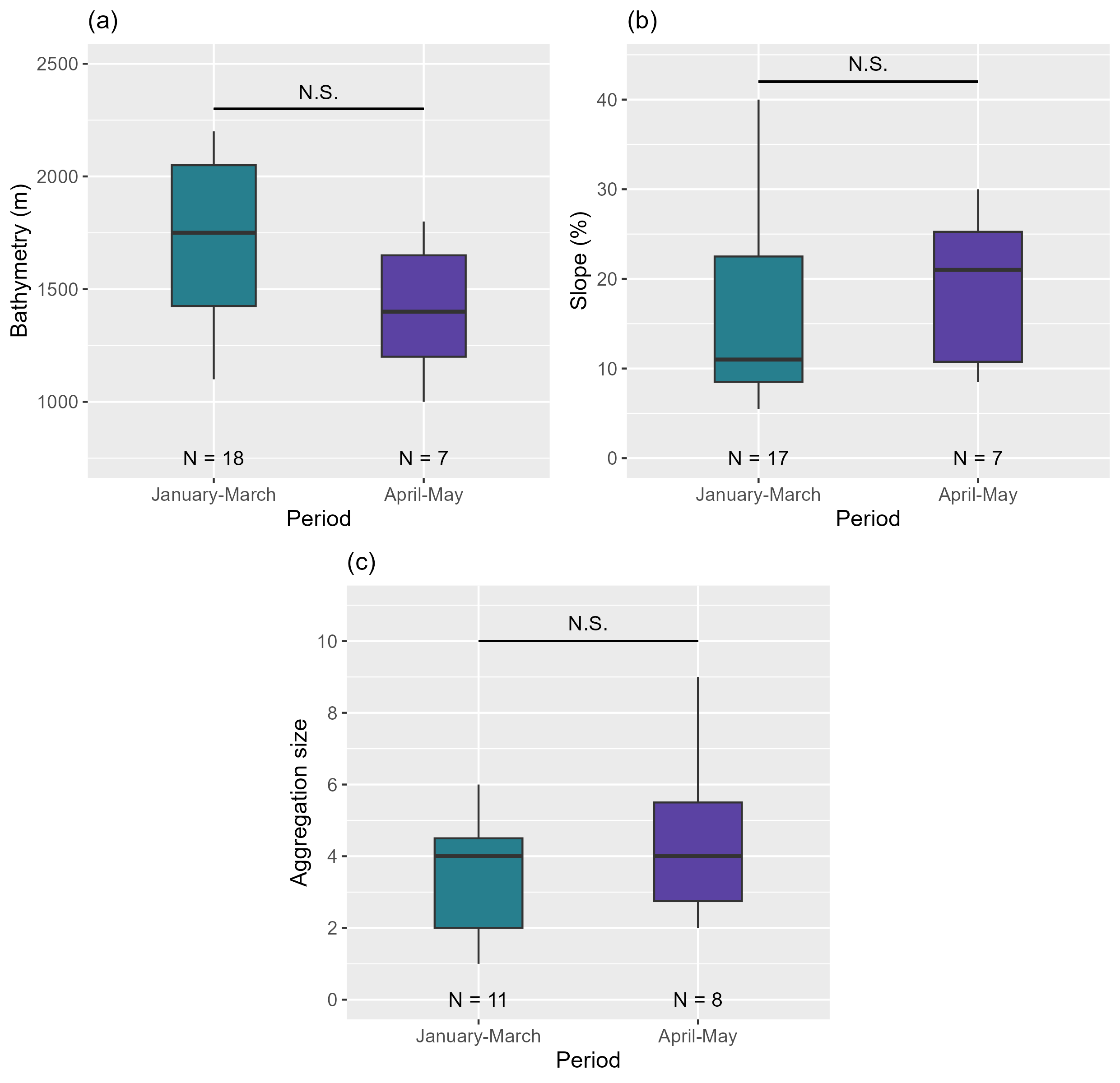

Supplement: Supplemental Information 12 [file peerj-13-19614-s012.zip › figure/Figure_S4.png]

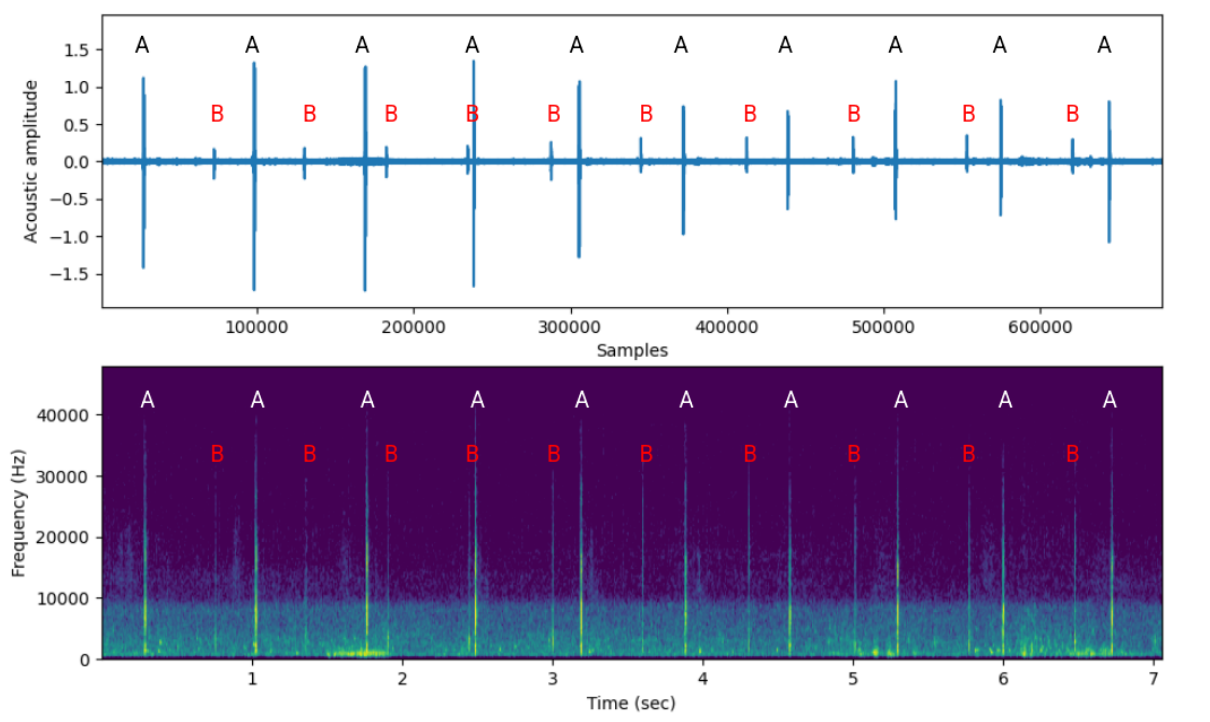

Supplement: Supplemental Information 12 [file peerj-13-19614-s012.zip › figure/Figure_S1.png]

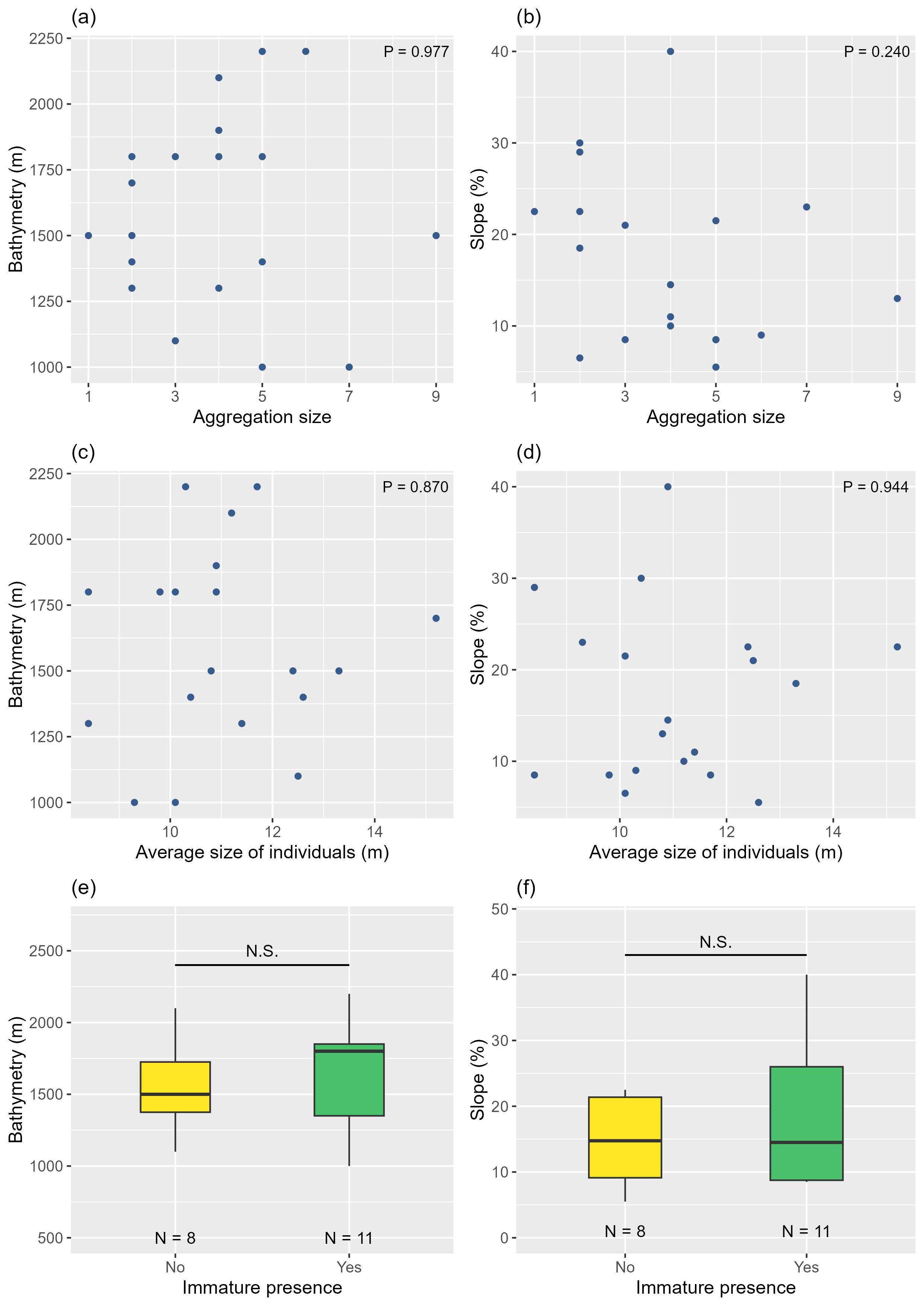

Supplement: Supplemental Information 12 [file peerj-13-19614-s012.zip › figure/Figure_S3.png]
